# Progress in Surgery

**Published:** 1896-07-18

**Authors:** 


### Progress in Surgery.

### ABDOMINAL SURGERY.

**Drainage after Laparotomy** has become much less frequent. Czempin[1] divides the cases in which drainage may be indicated into two great groups. Firstly, operations upon non-infectious tumours, with unfavourable complications caused by the condition of the relations of the operation wound, such as intraligamentary new growths resulting in the necessarily extensive separation of the pelvic tissue; and, secondly, operations upon infective tumours, or those thought to be infective, and operations through injury to the intestine, the escape of pus, or decomposed fluids. In the first group he is doubtful whether drainage does good, and in the second he is inclined to believe that if virulent organisms are present it can scarcely save life, and if the virulence is extinct it is not necessary. Grandin[2] speaks against the employment of glass tubes in drainage. He has given up the use of iodoform gauze because idiosyncrasy, which cannot be determined beforehand, not infrequently leads to poisoning. Where packing, as well as subsequent drainage, is the indication, he uses a long strip of plain, sterilized gauze about three inches wide. Where simple drainage is the aim, the ordinary sterile candle-wick or lamp-wick answers admirably.

**Shock after Abdominal Section** is regarded by Boise[3] not as a paresis of the nervous system, as has been usually claimed, but rather that it is an excessive irritation of the entire sympathetic nervous system, the result chiefly of an excessive stimulation of the vaso-motor nerves. This results in over-stimulation of primary vaso-motor and cardio-innervating centres causing a primary rapid and weak action of the heart. At the same time it excites an undue contraction of the arterioles, and causes the blood to be rapidly driven over into the veins, which thereupon progressively expand, thus preventing the blood from returning to the arterial system as rapidly as it should. Nutrition is therefore interfered with, and the nervous system more or less profoundly depressed. From such deductions he concludes that the following methods should be adopted in the treatment of surgical shock: (1) Inhalation of nitrate of amyl, not only while the patient is on the operating table, but repeated afterwards at intervals. (2) The hypodermic injection of nitro-glycerine in large doses, one-fifteenth to one-twentieth of a grain, repeated until the effect on the pulse is evident, should be the rule. (3) Repeated injections of hot saline solution, given by high enema, so that the fluid will pass into the transverse colon, are most valuable adjuvants, not only tending to relieve vaso-motor spasm, but also supplying to the circulation the fluid lost by hæmorrhage during operation. (4) Hypodermic injections of strychnia (one-twentieth to one-fifteenth of a grain) assist markedly, and an injection of morphia may be given to advantage immediately before operation.

**The Structure and Absorption Power of the Peritoneum** has been the subject of experiments by Muscatello,[4] who concludes that: (1) The diaphragm is the only portion of the serosa which is designed for the absorption of granular substances. This absorption takes place with extreme rapidity. (2) There exist in the abdominal cavity continuous fluid currents directed towards the diaphragm. It is the function of the mediastinal lymph glands to collect the lymph originating in the abdominal cavisy. (3) The endothelial cells of the serosa possess in part long processes. Under normal conditions they form a uniform layer possessing no openings. The formations described as stigmata or stomata are accidental products. At many points rounded spaces, which are covered on the surface by the lamella superficialis, may originate by the retraction of the protoplasm of two or three neighbouring cells. Under ordinary circumstances leucocytes are found here and there between the endothelial cells. (4) The limiting membrane, which is perforated in the district of the peritoneum diaphragmaticum, shows no trace of such openings at many other localities. (5) Finely granular substances (carmine) and soft pliable bodies (red blood corpuscles) penetrate the endothelial layer of the diaphragm in great part in a free state by pushing their way between the endothelial cells, in less part as inclusions of leucocytes. Large, firm bodies (starch) pass through in much the same manner, but the transmission of the largest of these granules takes place through the intervention of leucocytes, which spread themselves over the surface of the foreign body, thus enclosing it in a contractile layer.

Stickler[5] has also conducted experiments of somewhat similar nature on guinea pigs, and, allowing for any differences of susceptibility which may exist between guinea-pigs and man, it would seem from these experiments that fæcal matter, pus, perspiration, and urine are not so likely to cause trouble when introduced accidentally or purposely into the abdominal cavity as has been supposed. Carpet tacks introduced into the peritoneal cavity were covered with lymph and appeared to cause no mischief.

**The Treatment of Glenard's Disease by Abdominal Section** has been brought forward by Treves,[6] who states that the abdominal ptosis depends, in the main, upon a relaxation of the abdominal wall and of the supporting ligaments of the viscera, as a result of which the more conspicuous organs are found to have dropped to a lower level in the abdomen. It is usually met with in women, and has been ascribed to repeated pregnancies, undue exertion, and injuries. It is stated that the right bend of the transverse colon is the first part to descend, and this, in due course, is followed by the stomach, liver, kidneys, and other organs. As a result of this general ptosis we find certain asthenic symptoms, general depression, and ill-health. The patient—generally a woman—is unfit for exertion and only comfortable when recumbent. There is a sense of "weight" and "dragging" in the abdomen, a sense of burning in the epigastrium, vomiting, loss of appetite, and symptoms of dyspepsia. Colic is common. The kidneys are found to be moveable, the liver and stomach are prolapsed, and return, more or less, to their normal position when the patient is recumbent. In such a case Treves opened the abdomen and secured the liver in place by three stout silk sutures. The most important stitch was passed through the liver near its edge and penetrated the round ligament. The others involved the round ligament and the falciform ligament. Above the sutures were passed through the fibrous structures of the parietes by the side of the xyphoid cartilage, The patient made an excellent recovery, and her symptoms were relieved.

**Abdominal Injuries without External Wounds** present much difficulty in diagnosis, for Bryant[7] has shown that serious intra-abdominal lesions may occur without markedly grave symptoms, and conversely great collapse and urgent symptoms may occur in otherwise trivial injuries. Bryant endeavours to point out the circumstances under which the surgeon is justified in interfering. He considers that when, in any individual case, any one of the general symptoms, and particularly that of collapse, persists or becomes more marked, or when for a time the symptoms seem to have ameliorated and in a few hours again become grave, the conclusion that serious intra-abdominal injury has been sustained is right, and more particularly when the force which is supposed to have brought about the injury was of such a nature as to have been likely to have caused the mischief. Under such circumstances it is also probable that a solid viscus has been lacerated. If also under similar circumstances vomiting persists, or having ceased returns and is obstinate, serious trouble is suggested; if also blood appears in the renewed vomit and was absent in the primary, and its presence continues, rupture of the small intestine seems probable and most likely in the jejunum. Crushed kidney rarely requires surgical interference, which is only exceptionally applicable. Rupture of the liver or spleen kills generally by hæmorrhage, and when the laceration is severe the patient rarely recovers from the primary collapse sufficiently to justify surgical interference. The typical case for operative treatment is that of ruptured intestine, which may be considered highly probable after the receipt of a diffused injury to the abdomen, or a severe local injury, particularly if, as an immediate result of the accident, there is but little collapse, and where vomiting soon becomes a prominent and persistent symptom, with lasting local pain and great thirst, and with or without abdominal enlargement. With the presence of such symptoms, especially if they have persisted for eight or ten hours, a medium laparotomy may be performed. This measure, to prove successful, must be undertaken early, that is, not later than twenty-four hours after the receipt of the injury, and earlier if possible—for death within thirty-six or forty-eight hours of the accident is the rule. In old or feeble subjects such a measure ought not to be undertaken.

**Abdominal Sepsis treated by Intra-Venous Serum Injections** by Berlin,[8] Segond,[9] and others seems to hold out some hope in these serious cases. Berlin's case, which followed vaginal hysterectomy, appeared to be dying on the fourth day. The belly was distended and painful, and there was marked sub-normal temperature and frequent vomiting. An intra-venous injection of about a pint of artificial blood serum was given, and shortly afterwards repeated. Marked cyanosis and increased gravity of symptoms followed at once, but soon a decided improvement took place, and the patient was convalescent on the twelfth day of her illness. Segond[10] has had similarly favourable results.

[1] New York Med. Rec., Feb. 8th, 1896. [2] Amer. Med. Surg. Bullet., New York, Feb. 15th, 1896. [3] Ibidem, April 18th, 1896, p. 514. [4] Ibidem, Feb. 29th, 1896. [5] Med. Rec., New York, Feb. 1st, 1896, p. 156. [6] Brit. Med. Journ., Jan. 4th, 1896. [7] Lancet, Jan. 11th, 1896, p. 87 [8] Therapeut. Woch., No. 21, 1896; and Therapeut. Gaz., April 15th, 1896. [9] Ibidem. [10] Op. cit.

